# Adhesion and polarity-driven morphogenesis: Mechanisms and constraints in tissue formation

**DOI:** 10.1371/journal.pcbi.1013939

**Published:** 2026-06-22

**Authors:** Yoshiyuki T. Nakamura, Chikara Furusawa, Kunihiko Kaneko

**Affiliations:** 1 Department of Physics, The University of Tokyo, Tokyo, Japan; 2 Universal Biology Institute, The University of Tokyo, Tokyo, Japan; 3 Center for Biosystems Dynamics Research, RIKEN, Kobe, Japan; 4 Niels Bohr Institute, University of Copenhagen, Copenhagen, Denmark; Pázmány Péter Catholic University: Pazmany Peter Katolikus Egyetem, HUNGARY

## Abstract

Embryonic development in multicellular organisms exhibits diverse morphogenetic patterns, which can generally be categorized into fundamental types such as monolayer and multilayer spheres, as well as cell masses. Furthermore, we identify two distinct processes for the formation of spherical structures. These basic patterns are thought to be governed by the microscopic properties of intercellular adhesion. However, the specific mechanisms linking the microscopic factors to the emergence of distinct macroscopic morphogenetic patterns remain poorly understood. In this study, we explore how different morphogenetic patterns arise by employing a computational model that incorporates intercellular adhesion and polarity. Our results demonstrate that all fundamental morphogenetic patterns can be generated through the interplay of two key parameters: the polarity strength of the cell and the regulation of polarity via mechanical signals. Furthermore, analytical considerations reveal key mechanisms underlying the formation of these patterns. These findings highlight the critical role of physical constraints in morphogenesis and suggest potential applications to the design of artificial tissues and organoids.

## Author summary

Living organisms build their bodies through morphogenesis, during which cells autonomously arrange themselves into functional structures such as sheets, tubes, and spheres. From simple monolayered spheres to complex multilayered tissues organized by adhesion, it remains unclear how such diverse forms arise. Here, we mathematically modeled a population of proliferating cells governed only by two microscopic factors: the polarity strength of the cell and the time scale at which polarity is regulated by cell-cell contact. Surprisingly, we found that this minimal model reproduces five basic morphological types observed in living embryos, including monolayer/multilayer structures and two distinct modes of cavity formation: by wrapping around or by inflating from the inside. Systematic simulations revealed that these macroscale outcomes are determined solely by two parameters controlling polarity strength and its regulation, suggesting that simple physical rules underlie diverse developmental architectures. Analysis of the model uncovers phase transitions between the five morphogenetic types and reveals how varying polarity and adhesion can recapitulate features of real embryogenesis. Our work proposes a unified framework that connects microscopic polarity mechanics to diverse developmental morphologies and provides a foundation for future applications in organoid design and tissue engineering.

## 1. Introduction

**Fig 1:**
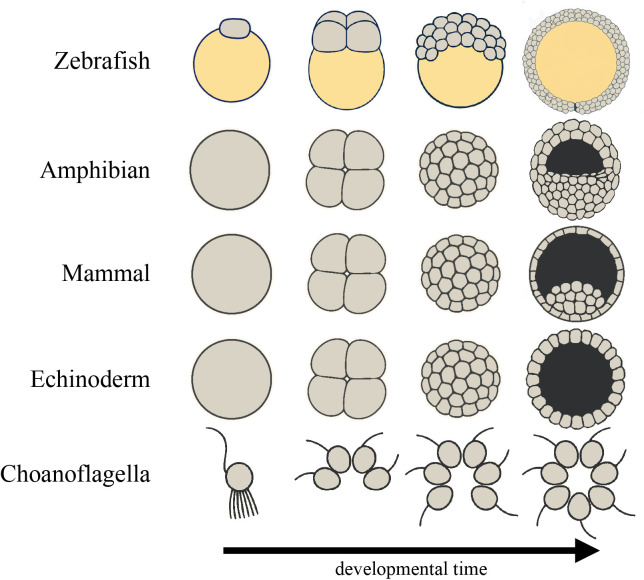
Illustrated comparison of early embryogenesis across metazoan lineages and colony formation in choanoflagellates. Gray regions represent cells, while yellow regions indicate yolk. The horizontal axis represents developmental time.

The development of multicellular organisms begins with a single cell, which proliferates and organizes into diverse macroscopic structures. Despite this shared starting point, the manner in which early embryonic cells arrange themselves varies significantly across different species. In zebrafish embryos, multiple cell layers form as the blastoderm spreads over the large yolk mass, a process characteristic of discoidal cleavage (Fig 1) [1–3]. In amphibian embryos, cells generate a multi-layered sphere by inflating from within, with differing cell layer numbers between the animal and vegetal poles (Fig 1) [1,4]. Mammalian embryos initially form a compact solid cluster, which later reorganizes into a blastocyst consisting of an outer monolayered epithelium and an inner cell mass [1,5]. Echinoderm embryos, by contrast, develop into a monolayered hollow sphere (Fig 1) [1,6]. Notably, even in choanoflagellates, the closest unicellular relatives of animals, cells form a monolayered sphere, involving the closure of an initially arc-shaped configuration (Fig 1) [7,8].

**Fig 2:**
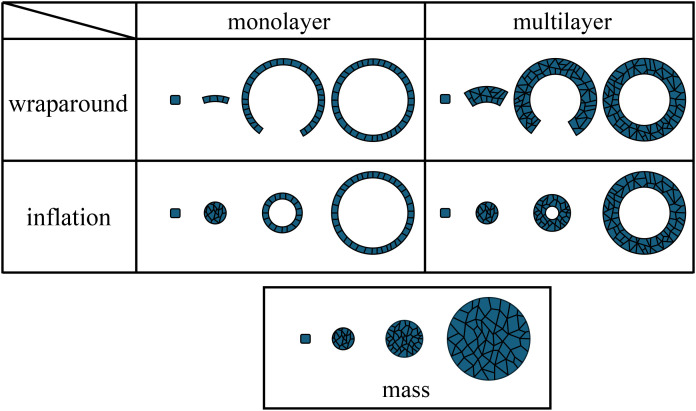
Categorization of the 5 basic types. The horizontal axis represents developmental time.

As observed in a variety of early embryos, we note that there are basic typical behaviors: cells form either masses or hollow spheres. When forming a sphere-like structure, the cell layer on the surface can be either single-layered or multi-layered in which the number of layers can vary. Additionally, in the case of sphere formation, there are two types: either a sphere is formed by wrapping around or it is formed by inflating from the center core cells. Thus the behaviors can be categorized into 2×2 classes of mono/multi-layered and wraparound/inflation, in addition to the mass formation case (Fig 2). We refer to these 5 classes as the “basic types.”

The diversity in morphogenetic patterns among these organisms does not appear to correlate with their phylogenetic relationships. This suggests that the mechanisms responsible for their differences may not be solely determined by genetic relatedness. Can we understand the basic types of morphogenesis as generic physical mechanisms [9–11]? What mechanisms contribute to generating distinct morphogenetic processes? One of the main drivers of morphogenesis is the sequential arrangement of individual cells while maintaining adhesion [9]. Cell adhesion is primarily mediated by adhesion proteins, with cadherins being a well-known example, which are a class of transmembrane proteins that facilitate cell-cell adhesion by forming adherens junctions [12]. Cadherins interact with catenins, which link the cadherins to the actin cytoskeleton, thereby providing mechanical stability and signaling functions [13]. The differential expression of cadherins between cell types can lead to the segregation of cell populations and the formation of distinct tissue structures [14]. For example, E-cadherin is crucial for the formation of epithelial layers, while N-cadherin is involved in the development of neural tissues [15].

Another important factor in cell-cell interaction is apico-basal polarity, which plays a crucial role in determining the mode of adhesion and, consequently, morphogenesis [16,17]. Apico-basal polarity refers to the spatial differences in the distribution of cellular components along the apico-basal axis of epithelial cells. This polarity is essential for the formation of proper epithelial tissues, as it is a dominant factor in determining the orientation of cells and formation of distinct cellular domains. The establishment and maintenance of apico-basal polarity are regulated by a complex network of polarity proteins, including the PAR complex, which interacts with cell adhesion molecules to coordinate cell positioning and tissue architecture [18]. Importantly, cell-cell adhesion is crucial for the establishment of apico-basal polarity by organizing the spatial distribution of polarity proteins, microtubules, and actin filaments, and coordinating their interactions with the cytoskeleton and cell membrane components [19,20]. Therefore, understanding the interplay between cell adhesion and polarity is essential for elucidating the mechanisms of morphogenesis.

Conceptually, the premise that cell adhesion and apico-basal polarity act together to give rise to cavities within tissues has been recognized; for instance, earlier physico-evolutionary arguments demonstrated that a hollow configuration naturally emerges from adhesive packing if cells are less adhesive on one polarized side [11]. Building on this foundation, several theoretical models of morphogenesis that consider the mechanical effects of polarity and adhesion have been proposed. For example, there are models that recapitulate the formation of a monolayer sphere by adding polarity to the cellular Potts model [21–23] or the cellular vertex model [24–27]. Additionally, cell-based models including polarity-dependent adhesion are also used to discuss the origin of the complexity of shapes [28] and robustness [29], from which the influence of polarity on the formation of morphology has been investigated. Previous theoretical studies have also highlighted the importance of nonequilibrium growth in epithelial morphogenesis. In particular, Cerruti et al. used a three-dimensional Potts-based model and experiments to show that the ratio between cell division and mechanical/lumen relaxation timescales determines whether epithelial growth proceeds close to equilibrium or far from it, and that rapid nonequilibrium growth can induce aberrant multilumen phenotypes [30]. In the present study, by contrast, we focus on the complementary regime in which cell division is sufficiently slow compared with positional and polarity relaxation, in order to extract the morphology-selection mechanism attributable specifically to polarity-dependent adhesion.

Of course, the actual morphogenesis involves complicated processes. Still, it will be important to explore how much of morphogenesis, in particular the five basic types, can be understood just by the adhesion and polarity. So far, however, consequences of these two factors for macroscopic morphogenetic patterns are poorly understood. In this study, we investigate the potential outcomes that emerge when considering only adhesion and polarity as the first step toward understanding the difference in morphogenesis among species. Following the spirit of statistical physics that links differences in macroscopic phases to variations in microscopic properties, we classify the basic types of morphogenesis that arise as distinct “phases” from differences in cell-associated parameters. Specifically, variations in cell polarity and adhesion strength may serve as critical determinants of these types. Furthermore, it aims to examine the mechanism of how the differences in microscopic factors give rise to different basic types of morphology. This approach provides a framework to connect micro-level cellular parameters with macro-level developmental outcomes, offering novel insights into the principles underlying tissue organization.

Here, for simplicity, we consider possible patterns of morphology formed only by adhesion among cells in a homogeneous population without given specific boundary conditions. Our model consists of a population of cells that proliferate while following the potential for adhesion and the dynamics of polarity control. We do not consider cell differentiation, to focus on only the physical force by adhesion and polarity, as a first step to understand the basic types of morphogenesis. By changing the strength of the polarity and the regulation of the polarity, we study how the morphogenesis process is altered. In addition, we uncover why the number of layers and the formation process differ by using simple analytical calculation.

From our results, we suggest that the number of layers and the formation process can be explained by the difference in adhesion. This result may also be applied to the design of organoids and other artificial tissues.

## 2. Model

We propose a model for the morphogenesis of a cell population. In this model, cells proliferate while following the potential for polarity-dependent adhesion and the dynamics of polarity control. Regarding cell polarity, we consider only one type here, assuming the apico-basal polarity as in epithelial cells. It should be noted that other types of polarity, such as planar cell polarity, are not considered here.

In addition, we primarily consider the case of two-dimensional space. The model can be extended to three dimensions, as discussed in Section 3.5.

### 2.1. The dynamics of cell position and polarity

**Fig 3:**
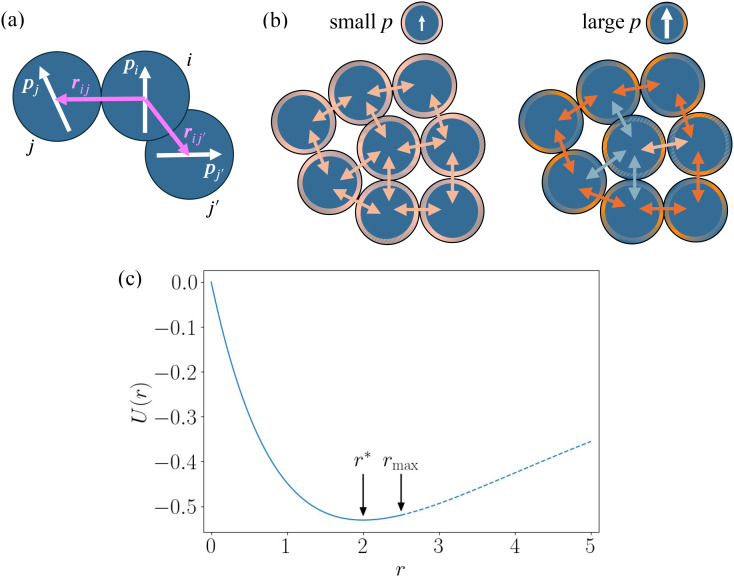
(a) Schematic representation of the model. Cells are represented as blue circles. All cells have the same typical radius $r^{*}/2$, which is set to be 1. Each cell has an intrinsic polarity vector (white arrows). Cells move while interacting with each other. Their dynamics depend on both polarity and the relative position. (b) Schematic representation of cell populations with small *p* (left) and large *p* (right). Coloring on the cell periphery represents the local adhesivity determined by the term $p_{i}\times {\hat{r}}_{ij}$, where dark orange, light orange, and blue indicate high, medium, and low values, respectively. This gradient illustrates the degree of adhesion anisotropy within each cell. Double-headed arrows indicate the magnitude of the pairwise adhesion factor $S_{ij}$ between cells; the color of the arrows (from dark orange to blue) indicates the strength of the adhesive interactions from strong to weak. (c) The shape of the function *U*(*r*). It takes a minimum at $r^{*}=2$ (i.e., the radius of the cell is 1). In the region where *r* > *r*_max_ = 2.5, there is no interaction, which is indicated by the dotted line.

Each cell has polarity, which is represented as a vector of magnitude *p* (Fig 3a).


$$\begin{array}{c} p_{i}=(p\cos\theta_{i},p\sin\theta_{i}{)}^{T} \end{array}$$ (1)

Here, the superscript T denotes transpose. In this model, two cells interact with each other through adhesion. The adhesion is given by short-ranged interaction where there is a cutoff distance of the interaction, which will be discussed later. When two cells interact, they follow dynamics which depend on those positions and polarities. Representing the positions of cells *i* and *j* by $r_{i}$ and $r_{j}$ respectively, and the distance between them by $r_{ij}$, the potential $V_{ij}$ for adhesion force between cells *i* and *j* is described as


$$\begin{array}{c} V_{ij}=S_{ij}\cdot U(r_{ij}). \end{array}$$ (2)

Here, *S* is a factor dependent on polarity, and *U* is a distance-dependent function, which are given by


$$\begin{array}{c} S_{ij}=(p_{i}\times {\hat{r}}_{ij})\cdot (p_{j}\times {\hat{r}}_{ij})+1, \end{array}$$ (3)

$$\begin{array}{c} U(r_{ij})=e^{-r_{ij}}-e^{-r_{ij}/\beta } \end{array}$$ (4)

where $r_{ij}=r_{j}-r_{i}$ and ${\hat{r}}_{ij}=r_{ij}/r_{ij}$. As can be seen from the equations, $S_{ij}$ is maximized when the polarity of cell *i* and that of cell *j* are in the same direction and perpendicular to ${\hat{r}}_{ij}$. In this model, *p* sets the adhesive differential between the most and least adhesive parts of the cell surface, as illustrated in Fig 3b. For simplicity, we refer to this parameter *p* as “polarity strength” throughout this manuscript. The factor $S_{ij}$ ranges from $1-p^{2}$ to 1 + *p*^2^, depending on the relative orientation between the polarity axis and the cell-cell contact. This interpretation is useful for understanding why changes in *p* affect whether a cavity forms. The formation of a cavity can be understood as a competition between the gain in adhesive energy and the cost of maintaining non-adhesive surfaces. When *p* is large, the adhesive contrast is high enough that the cells prefer to maximize contacts at their highly adhesive lateral sides. This radial alignment creates a “non-adhesive core” that prevents the collapse of the structure into a solid mass, thereby stabilizing a central cavity.

The offset term +1 in Eq. (3) provides a baseline contribution to adhesion and, since we restrict the polarity strength to p≤1 in this study, keeps the polarity-dependent factor $S_{ij}$ non-negative. As a result, polarity modulates the strength of adhesion between cells, rather than changing the interaction from attractive to repulsive. In this sense, $S_{ij}$ describes a difference in adhesive strength between more and less adhesive regions of the cell surface. $U(r_{ij})$ is always negative and is minimized when the distance between cell *i* and cell *j* satisfies $U^{\prime }(r^{*})=0$ (Fig 3c). It should be noted that since the value of $r^{*}$ does not depend on the value of $S_{ij}$, all cells in this model can be approximated as circles with a radius of $r^{*}/2$. For $r<r^{*}$, dU/dr<0, so that there is a repulsive force between cells, and for $r>r^{*}$, dU/dr>0, so that there is an attractive force towards the equilibrium distance $r^{*}$. This force should be short-ranged, and here, we introduced a cutoff distance *r*_max_ beyond which $U^{\prime }(r)=0$, as will be shown later.

In lowering this potential value $V_{ij}$, the polarity is aligned due to the effect of $S_{ij}$, and the radius of the cell approaches $r^{*}/2$ due to the effect of *U*. Here, we determine β so that the radius of the cell is 1, that is $r^{*}=2$.

As mentioned before, we set the maximum distance of interaction *r*_max_ to consider only local interactions (Fig 3c). In other words, when $r_{ij}$ exceeds *r*_max_, the interaction between *i* and *j* does not work. Let $N_{i}$ be the set of j(≠i) such that $r_{ij}<r_{\text{max}}$, then the potential $V_{i}$ applied to cell *i* is the sum of interactions with other cells $j\in N_{i}$.


$$\begin{array}{c} V_{i}=\sum_{j\in N_{i}}V_{ij}. \end{array}$$ (5)

Here, we set *r*_max_ = 2.5.

The position $r_{i}$ follows the overdamped dynamics using the above potential.


$$\begin{array}{c} \frac{\text{d}r_{i}}{\text{d}t}=-\tau_{V}\frac{\text{d}V_{i}}{\text{d}r_{i}}, \end{array}$$ (6)

where $\tau_{V}$ is a parameter that represents the time scale of the velocity.

On the other hand, the polarity $\theta_{i}$ is controlled not only by the potential $V_{i}$ but also by adhesion. Here we choose the dynamics


$$\begin{array}{c} \frac{\text{d}\theta_{i}}{\text{d}t}=-\tau_{V}\frac{\text{d}V_{i}}{\text{d}\theta_{i}}-\tau_{B}\sum_{j\in N_{i}}\left(\frac{\text{d}\left({\hat{p}}_{i}\cdot {\hat{r}}_{ij}\right)}{\text{d}\theta_{i}}\right), \end{array}$$ (7)

where the second term represents the polarity control with the time constant $\tau_{B}$. Here, ${\hat{p}}_{i}$ is the unit vector of $p_{i}$. With this control, the polarity is directed so that the dot product of the position ${\hat{r}}_{ij}$ and polarity $p_{i}$ can be minimized. In other words, the polarity of cell *i* is forced to face the opposite direction to the adhesive surface. This term should be understood as a phenomenological polarity-reorientation rule. It captures the tendency for the apico-basal polarity to become oriented away from adhesive interfaces. Several experimental results support the fact that apico-basal polarity is controlled by mechanical stress and is often oriented perpendicular to the adhesion surface [19,20]. The validity of this assumption will be discussed further in the discussion section.

### 2.2. Cell division

In this model, we consider the cell division process to increase the number of the cells. This process is implemented by randomly selecting a single cell at a certain fixed interval ($\tau_{\text{div}}$) needed for cell division and dividing it. The new cell is generated at a position adjacent to the parent cell. That is, when a cell *i* divides, a new cell $i^{\prime }$ is randomly placed on the circumference with a radius of $r^{*}$ centered on the position $r_{i}$ of cell *i*. The polarity of the new cell $i^{\prime }$ has a random angle $\theta^{\prime }$. The daughter cell is initially placed at a random position adjacent to the parent cell. Then, its position and polarity subsequently evolve under the forces described by Eqs. (6) and (7), leading the daughter cell to quickly relax into an appropriate configuration.

In this study, we focus on morphogenesis driven by polarity-dependent adhesion. In the cell division method introduced above, a cell division process that is too fast compared to the dynamics of position and polarity is not considered. If the division is too fast, it always dominates over the effect of polarity. In fact, such rapid division just leads to the formation of a mass regardless of polarity or unstable phenomena such as buckling. These phenomena are beyond the scope of this study. Accordingly, the present analysis should be interpreted as applying to a slow-growth regime, in which local positional and polarity relaxation substantially proceeds before cell division. Our aim is to extract the morphology-selection mechanism due to polarity-dependent adhesion rather than nonequilibrium effects induced by excessively rapid proliferation. We will revisit the validity of this method in the discussion section. We will also discuss the issue of placement of daughter cells in the discussion section.

## 3. Results

The essential dynamics of our model arise from the interplay between adhesion and polarity. The polarity strength, controlled by *p*, determines how rigidly neighboring cells maintain their relative positions. In addition, the polarity vectors tend to orient in a mutually repulsive manner; the ratio $\tau_{B}/\tau_{V}$ governs this local polarity arrangement. This local polarity arrangement, in turn, constrains the emergence of global morphology. In the present analysis, we fix $\tau_{V}$ and vary $\tau_{B}$ to explore how polarity affects morphogenesis.

With this setup, we first show that the model can reproduce the types of morphologies introduced in the introduction by tuning *p* and $\tau_{B}$. We then provide an analytical estimate of the phase boundaries, followed by an extension of the model to generate more complex morphologies. Finally, we demonstrate that the model can be generalized to three dimensions, which does not substantially alter the two-dimensional results.

### 3.1. Typical behaviors

**Fig 4:**
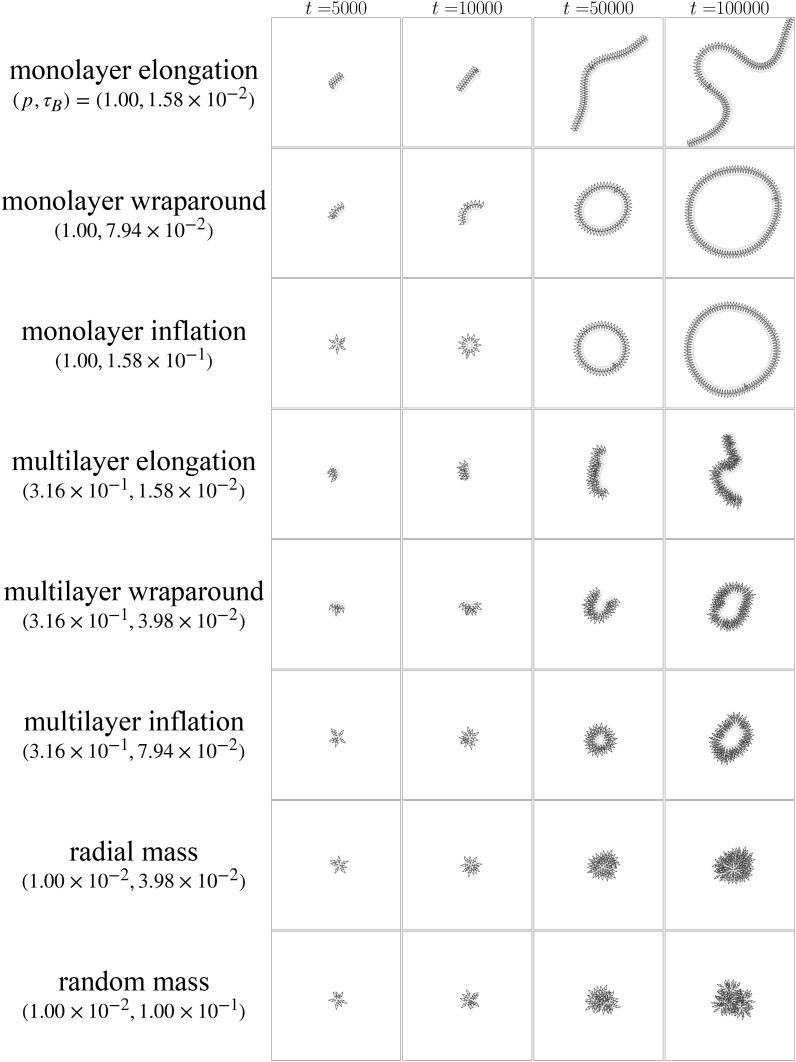
The time series illustrating typical eight behaviors: monolayer elongation, monolayer wraparound, monolayer inflation, multilayer elongation, multilayer wraparound, multilayer inflation, radial mass, and random mass. Each row corresponds to one phase, with the system parameters $\left(p,\tau_{B}\right)$ indicated below the corresponding labels. Circles represent the cells, and arrows represent the polarity.

First, we present some representative behaviors, which appear depending on the system parameters *p* and $\tau_{B}$ (See Fig 4). Note that all the results shown below are calculated up to $t=3\times 10^{5}$, and $\tau_{\text{div}}=10^{3}$. The initial cell is placed at the center of a square region with a width of 100 in both *x* and *y* axes. We adopt boundary conditions where each cell is confined within this square region. However, if even a single cell comes into contact with the edges of the region, it is not used in subsequent analyses, so these boundary conditions do not affect the main results.

#### Monolayer elongation.

Cells align in a row perpendicular to the polarity to form a monolayer. The newly generated cells enter the layer, and the layer extends horizontally. As the line grows longer, limited cell proliferation leads to the “buckling” instability to bend the layer at $t\approx 5\times 10^{4}$. This, however, is beyond our concern.

#### Monolayer wraparound.

When $\tau_{B}$ is slightly increased, the cells align in a row as in the previous section, but they extend with a certain curvature and finally stick together at the ends to form a circle.

#### Monolayer inflation.

When $\tau_{B}$ is further increased, the cells are still aligned in a row, but they inflate from a lumped state to form a circle.

#### Multilayer elongation.

When *p* is smaller than that of the monolayer cases, cells form multiple layers aligned with the polarity and extend horizontally as a whole.

#### Multilayer wraparound.

Even in the multilayer region, wraparound was observed.

#### Multilayer inflation.

When $\tau_{B}$ was further increased, inflation of a multilayer circle was observed.

#### Radial mass.

When *p* is much smaller, a mass aligned with the polarity radially is formed without a hole in the center.

#### Random mass.

When $\tau_{B}$ is large in the multilayer region, a mass with misaligned polarity is formed.

### 3.2. Robustness against cell-cell variability

In the analyses above, we mainly considered the homogeneous case, in which all cells share the same parameter values. To examine whether the observed morphologies are robust to cell-to-cell variations, we also simulated a heterogeneous version of the model where the parameter *p* or $\tau_{B}$ varied across cells. Specifically, the parameter value for each cell was drawn from a log-normal distribution, where the center was set to the baseline value of the homogeneous simulation, and the standard deviation was set to 10% of the grid size of the phase diagram. Under this heterogeneity, the representative behaviors corresponding to those in Fig 4 were qualitatively unchanged (see Figs. A and B in S1 Appendix for details). These results indicate that the basic morphogenetic types described here are robust to modest cell-to-cell variability.

### 3.3. Classification of the behaviors and the phase diagram of the model

**Fig 5:**
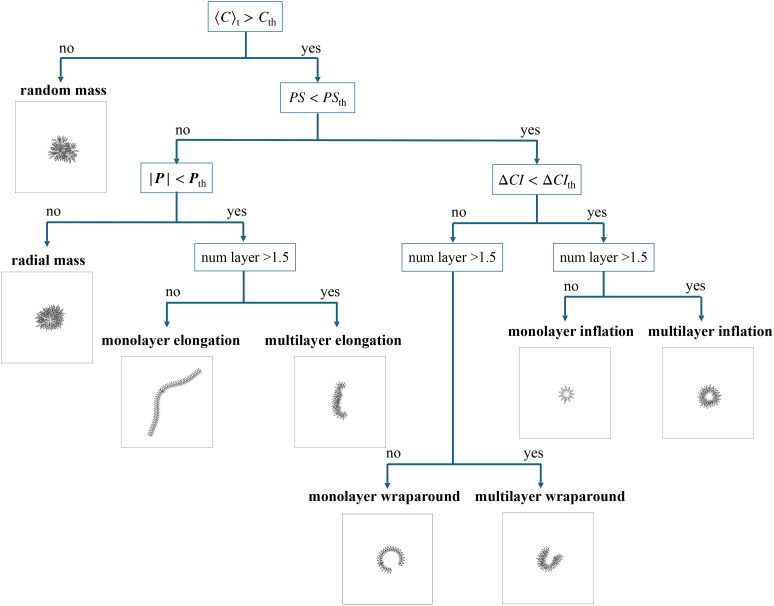
Flowchart of the classification of morphologies observed in this model. Representative behaviors of each morphology are shown under the names of the behaviors.

The morphologies observed in our model can be classified into eight distinct types. They are distinguished by four main criteria: (i) the degree of polarity alignment, quantified by the time-averaged polarity correlation; (ii) the presence or absence of a central hole and the manner in which it forms; (iii) the magnitude of the mean polarity vector, which reflects the extent of global polarity; and (iv) the number of layers in the resulting structure. A schematic overview of this classification is shown in Fig 5, along with representative snapshots of each type.

To make these criteria quantitative, we introduce the following quantities. The polarity correlation ($\langle C\rangle_{t}$) measures how strongly neighboring cells align their polarities over time. The persistence *PS*, calculated using the method of persistent homology, characterizes whether a stable hole is maintained within the structure. The mean polarity magnitude (|P|) indicates the degree of overall polarity order across the system. Finally, the change in circularity index at the moment of hole formation (ΔCI) distinguishes whether the hole grows by inflation or by wraparound closure. Details of the definitions and computational procedures of these quantities are provided in Section 2 in S1 Appendix.

Based on these quantities, the classification proceeds as summarized in Fig 5. Configurations with low polarity correlation are categorized as “random mass.” Among the remaining cases, the presence or absence of a hole is then evaluated by *PS*. Structures without a hole are further divided by the magnitude of the mean polarity vector: those with low |P| are termed “radial mass,” and those with sufficiently high |P| are classified into elongation types. The elongation types are further separated into “monolayer” and “multilayer” depending on the number of layers (with the boundary at 1.5). For hole-containing structures, the distinction between “inflation” and “wraparound” is based on ΔCI, and each of these is also subclassified into monolayer and multilayer cases depending on the layer number.

The results presented below were analyzed using the following threshold settings: *C*_th_ = 0.8, *PS*_th_ = 3, *P*_th_ = 0.3, and $\Delta CI_{\text{th}}=1$. These values were chosen to effectively separate the qualitatively different behaviors, and small changes in the thresholds do not significantly affect the classification.

**Fig 6:**
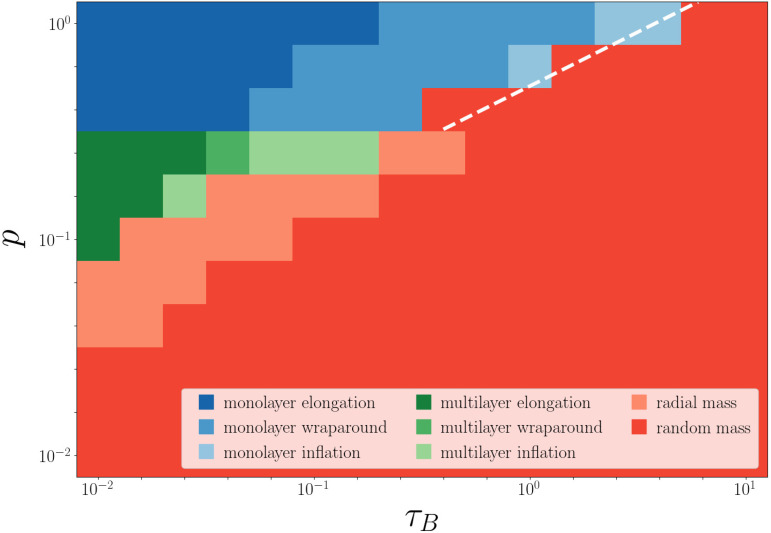
The phase diagram based on *p*, polarity strength, and $\tau_{B}$, the time scale of angle change. Each color represents the behavior shown in the legend. For each parameter set, 50 independent simulation runs were performed, and the most frequently observed behavior is shown by the color of the cell. The white line is obtained from the theoretical estimate of Eq. (18).

Summarizing these behaviors, we obtain the phase diagram shown in Fig 6. Each grid has 50 samples. We define the representative behavior of the grid as the one that appears most frequently in the 50 samples.

**Fig 7:**
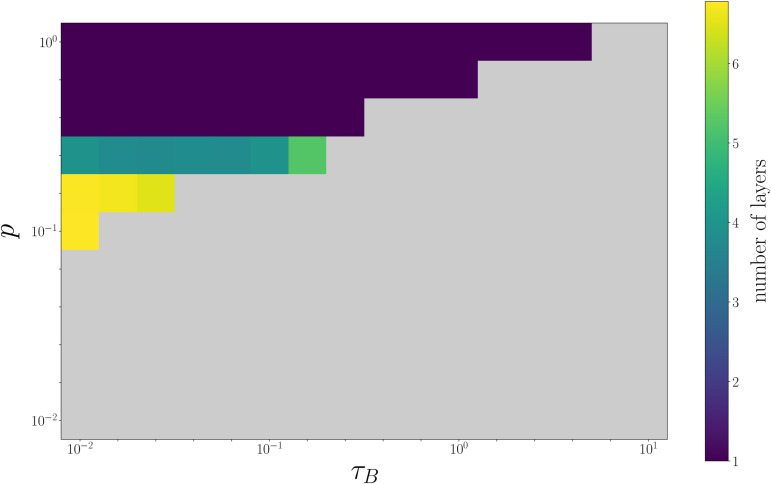
Dependence of the number of layers on *p* and $\tau_{B}$. The color of each grid represents the average number of layers observed, while the light gray regions correspond to the random-mass or radial-mass phases and are excluded from consideration.

First, monolayer and multilayer phases are separated by the magnitude of *p*, of p≈0.3. Within the multilayer phase at $\begin{array}{c} p≳0.3 \end{array}$, the number of layers decreases as *p* increases (Fig 7). In addition, wraparound and inflation are distinguished by the magnitude of $\tau_{B}$, although the boundary between them depends on *p*. The random mass phase appears when *p* is small or $\tau_{B}$ is excessively large. Note that wraparound is not observed when $\tau_{B}$ is small in the monolayer phase simply because the curvature is too small for the ends of the layer to connect before buckling occurs.

From this phase diagram, the parameter *p*, which controls the strength of cell-cell adhesion, determines whether cells form a monolayer or a multilayer. The second key parameter, $\tau_{B}$, controls how strongly neighboring cells tend to orient their polarity in opposite directions. As $\tau_{B}$ increases, the macroscopic dynamics undergo a transition from wraparound to inflation. Thus, wraparound phase results when $\tau_{B}$ is small, while sufficiently large $\tau_{B}$ values lead to inflation. If $\tau_{B}$ is too large, polarity is not oriented at all and the system collapses into the random mass phase.

The phase boundaries depend not only on $\tau_{B}$ but also on *p*. Moreover, inflation is also observed in the multilayer phase, but the required $\tau_{B}$ is much smaller than in the monolayer case. The random mass phase, in turn, appears either when adhesion is weak (*p* small) or when $\tau_{B}$ is excessively large.

To summarize, the collective behavior of cells with polarity-dependent adhesion falls into one of these eight categories depending on *p* and $\tau_{B}$. This result suggests that by considering only the magnitude of polarity and its repulsive effect on adhesive surfaces, basic morphogenetic patterns emerge, and no other patterns exist.

### 3.4. Analysis of phase boundaries

How do these differences in phases appear? To understand the reasons behind the phase diagram, we perform a bifurcation analysis by reducing the system to a few cells to study the transitions between mono-multi layers and wraparound-inflation in the monolayer phase.

#### Analysis of bifurcation between monolayer and multilayer.

In this subsection, we focus on the change between monolayer and multilayer in the phase diagram to understand it in terms of bifurcation.

**Fig 8:**
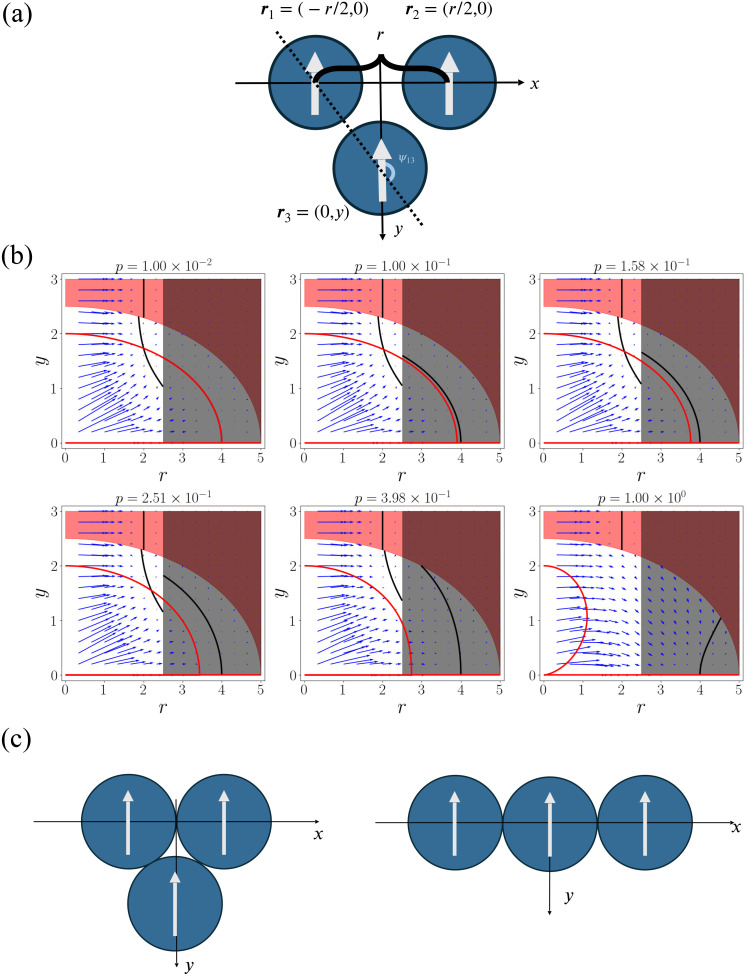
Three-cell bifurcation analysis of the monolayer–multilayer transition. **(a)** In this analysis, we consider a system of three cells interacting with each other, all having the same polarity direction. When cells 1, 2, and 3 are located as shown in the figure, we investigate how the initial positions determined by *r* and *y* influence the final configuration of the three cells from a dynamics perspective. **(b)** Differences in the dynamics of *r* and *y* and the resulting fixed points for each ***p*.** The blue arrows represent the velocity field, while the black and red lines represent the nullclines of *r* and *y*, respectively. The black and red shaded areas correspond to $r_{12}>r_{\text{max}}$ and $r_{13}>r_{\text{max}}$, respectively. **(c)** Left: Cell configuration at the fixed point $y\approx \sqrt{3},r\approx 2$. Right: Cell configuration at the fixed point *y* = 0, *r* = 4.

Here, we consider a system consisting of three cells with their polarities in the same direction. In this analysis, we set a line connecting the centers of cells 1 and 2 as the *x*-axis, and the midpoint of these two cells as the origin (Fig 8a). We examine if the cell 3 moves upward leading to a monolayer structure or not. Specifically, we analyze whether the third cell goes upward and is aligned with the first and second cells to form a one-dimensional chain in the stable state. From the symmetry of the interaction received from cells 1 and 2, the cell 3 does not move in the x-direction whereas it can move along the *y*-axis perpendicular to the *x*-axis. Then, the dependence of the cell configuration on the parameter *p* is studied in terms of bifurcation between mono- and multilayer.


$$\begin{array}{ccc} \frac{\text{d}y}{\text{d}t} & = & {\left(\frac{\text{d}r_{3}}{\text{d}t}\right)}_{y} \end{array}$$ (8)

$$\begin{array}{ccc} & = & -2\tau_{V}\cos\psi_{13}\left(\left(p^{2}\sin^{2}\psi_{13}+1\right)U^{\prime }(r)-\frac{2U(r)}{r}p^{2}\sin^{2}\psi_{13}\right) \end{array}$$ (9)

$$\begin{array}{ccc} \frac{\text{d}r}{\text{d}t} & = & {\left(\frac{\text{d}r_{2}}{\text{d}t}-\frac{\text{d}r_{1}}{\text{d}t}\right)}_{x} \end{array}$$ (10)

$$\begin{array}{ccc} & = & \left\{ \begin{array}{cc} -2\tau_{V}\left(2U^{\prime }(r)\right)-2\tau_{V}\left(\left(p^{2}\sin^{2}\psi_{13}\right)U^{\prime }(r)\cdot \frac{r}{2}+U(r)\frac{2}{y}p^{2}\sin\psi \cos^{3}\psi \right) & r_{12}<r_{\text{max}},r_{13}<r_{\text{max}}\\ -2\tau_{V}\left(\left(p^{2}\sin^{2}\psi_{13}\right)U^{\prime }(r)\cdot \frac{r}{2}+U(r)\frac{2}{y}p^{2}\sin\psi \cos^{3}\psi \right) & r_{12}\geq r_{\text{max}},r_{13}<r_{\text{max}}\\ -2\tau_{V}\left(2U^{\prime }(r)\right) & r_{12}<r_{\text{max}},r_{13}\geq r_{\text{max}}\\ 0 & r_{12}\geq r_{\text{max}},r_{13}\geq r_{\text{max}} \end{array}\right. \end{array}$$ (11)

Here, $\sin\psi_{13}=\frac{r/2}{\sqrt{(r/2{)}^{2}+y^{2}}},\cos\psi_{13}=\frac{y}{\sqrt{(r/2{)}^{2}+y^{2}}}$.

To analyze the system of (*r*,*y*), we consider two nullclines dy/dt=0 and dr/dt=0. Although analytic representation is difficult to obtain, they are drawn numerically as in Fig 8b. The result is shown in Fig 8b with the velocity field.

When the value of *p* is large (p≥0.5), this system has only one stable fixed point with *y* = 0 and *r* = 4. This means that all three cells are aligned within the same axis (Fig 8c). As *p* decreases, there is a saddle-node bifurcation at p≈0.3 so that this system with smaller *p* has two stable fixed points with (*y* = 0, *r* = 4) and $\left(y\approx \sqrt{3},r\approx 2\right)$. The latter fixed point means that the third cell is located below the first two cells (Fig 8c). The existence of this fixed point allows the system to form a multilayer. This bifurcation point at p≈0.3 roughly agrees with the boundary in the phase diagram in Fig 6. Note that the fixed point with *y* = 0 and *r* = 4 is still stable in this region.

Since the dynamics of the polarity *p* is neglected, this analysis does not strictly explain the entire boundary between monolayer and multilayer shown in Fig 7. In this derivation, we assumed that the polarity of the three cells is aligned, and the dynamics of p(θ) in the actual model is not considered. If the dynamics of p(θ) in the actual model is considerably different from this assumption, the analysis in this section is not valid. For example, in the monolayer-inflation phase where $\tau_{B}$ is large, the polarities of the three cells are radially aligned in the steady state of the three cells. This is beyond the scope of the discussion in this section. However, in the region where $\tau_{B}\leq 0.2$, this assumption on aligned polarity is valid to some extent, according to the numerical observation. Therefore, at least for the boundary between monolayer and multilayer regions, the present estimate of phase boundary is valid.

#### Analysis of the boundary between monolayer wraparound and inflation.

**Fig 9:**
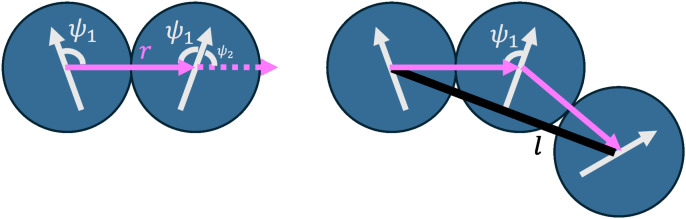
Two-body reduction of a three-cell system for the wraparound–inflation boundary. In this analysis, we focus on whether the two end cells interact with each other when three cells are aligned while maintaining the polarity angles of the two-cell system. The angles of the polarity vectors with respect to *r* in the two-cell system are denoted as $\psi_{1}$ and $\psi_{2}$ in the figure. Due to symmetry, $\psi_{2}=\pi -\psi_{1}$. The distance between the two end cells is represented by the black line, and its length is denoted as *l*.

Next, we analyze the phase boundary between wraparound and inflation, in terms of cell-cell interactions. We focus on the change between wraparound and inflation in monolayer case [31]. Here, we consider a three-cell system, and examine whether the cells at both ends interact or not. This is because when the cells at both ends do not interact, a system with more than three cells can be decomposed into consecutive adjacent two-cell systems, leading to angles between them needed for a wraparound structure. Now, assume that the ends do not interact with each other. In this case, the three-body system can be represented by two adjacent two-body systems (Fig 9). The fixed points of a two-body system can be derived as follows.


$$\begin{array}{c} \frac{\text{d}\theta_{i}}{\text{d}t}=-\tau_{V}\frac{\text{d}V_{i}}{\text{d}\theta_{i}}-\tau_{B}\frac{\text{d}\left({\hat{p}}_{i}\cdot {\hat{r}}_{ij}\right)}{\text{d}\theta_{i}}=0 \end{array}$$ (12)

Since the two-body system is described by two degrees of freedom $\theta_{1}$ and $\theta_{2}$, by solving Eq. (12) for $\theta_{1}$ and $\theta_{2}$, we get


$$\begin{array}{c} -\tau_{V}U(r^{*})p^{2}\sin\psi_{1}\cos\psi_{1}+\tau_{B}\sin\psi_{1}=0, \end{array}$$ (13)

$$\begin{array}{c} \sin\psi_{1}(-\tau_{V}U(r^{*})p^{2}\cos\psi_{1}+\tau_{B})=0. \end{array}$$ (14)

Therefore, Eq. (12) holds when $\psi_{1}$ satisfies either of the following conditions.


$$\begin{array}{c} \left\{ \begin{array}{c} \sin\psi_{1}=0,\\ \cos\psi_{1}=\frac{\tau_{B}}{\tau_{V}U(r^{*})p^{2}}. \end{array}\right. \end{array}$$ (15)

When these two-body systems are connected, the distance between the ends can be calculated. Therefore, assuming the stable solution [32], the condition that the ends of the cells do not interact with each other is written as


$$\begin{array}{c} l=2r^{*}\sin\psi_{1}\geq r_{\text{max}}=2.5 \end{array}$$ (16)

$$\begin{array}{c} 2r^{*}\sqrt{1-{\left(\frac{\tau_{B}}{\tau_{V}U(r^{*})p^{2}}\right)}^{2}}\geq 2.5. \end{array}$$ (17)

Substituting the values used in the simulation $r^{*}=2$, $U(r^{*})\approx -0.5$, and $\tau_{V}=10$ into the equations, we get:


$$\begin{array}{c} \frac{\tau_{B}}{p^{2}}\leq 3.90\ldots \approx 4 \end{array}$$ (18)

Within the range of the above inequality, a wraparound is expected to occur. This can explain the trend of the boundary between wraparound and inflation in the phase diagram (Fig 6).

### 3.5. 3D version of the model

So far, we have presented the results for the two-dimensional case. However, the model can be straightforwardly extended to a three-dimensional case. In the three-dimensional case, we also confirmed the same types of morphogenesis as those observed in the two-dimensional model, except for the random mass phase. These are shown in Supporting Figures (Fig N to T in S1 Appendix). In three dimensions, there is one more degree of freedom regarding the direction of polarity compared to two dimensions, which is expected to amplify the polarity alignment so that random mass phase will be unlikely to exist. Except for this, we have the seven phases as observed in the two-dimensional case, with similar dependence on *p* and $\tau_{B}$.

### 3.6. Possible extension of the model to correspond to actual morphogenesis

In the previous sections, we demonstrated that basic types of cell configuration (Fig 6) are represented as distinct “phases” with the parameters for adhesion and polarity. In the real developmental process, these parameters are not necessarily identical over all cells, but can depend on the position or on their cell types. In this section, we show that this model can express more complex morphologies by combining the results of each phase. We show that the early embryos of fish and mammals are reproduced by simply introducing the spatial gradient of $\tau_{B}$ or time evolution of *p*.

#### Gradient of $\tau_{B}$ along y-axis: as a possible model for fish morphogenesis.

**Fig 10:**
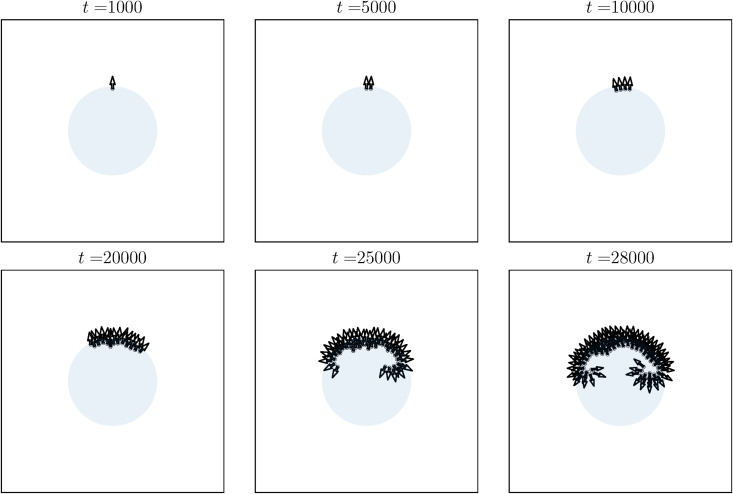
Morphogenesis process with a gradient in $\tau_{B}$. Here, the polarity-regulation coefficient follows $\tau_{B}=2-0.1(y_{i}-50)$.

In the early embryos of fish, cells generally form a multilayer wraparound. However, within this process, cells at the leading edge are drawn inward as they are engulfed [1]. This behavior can be reproduced by assuming that each cell has a different $\tau_{B}$ value depending on its position. As shown in Fig 10, by setting a gradient in $\tau_{B}$ along the *y*-axis, the effective curvature radius becomes smaller in regions with high $\tau_{B}$, allowing the leading edge cells to be drawn inward [33]. This corresponds to the characteristic morphogenetic movement in fish epiboly, where blastodermal cells undergo inward migration while simultaneously advancing over and enclosing the yolk.

#### Time-dependent p driven by a diffusive signal: as a possible model for mammalian morphogenesis.

**Fig 11:**
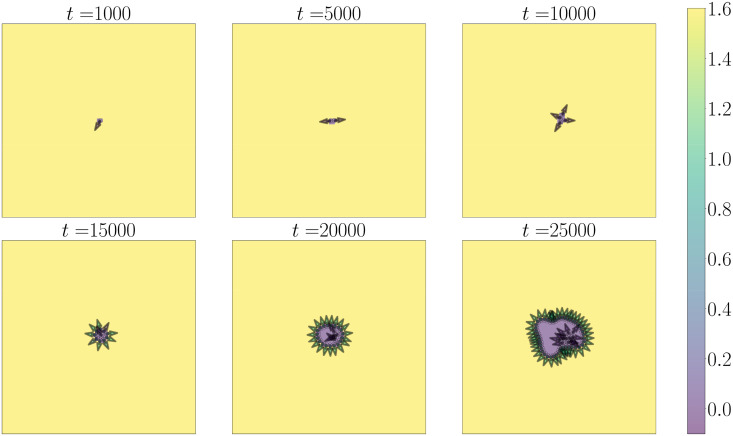
Morphogenesis process when *p* develops over time due to a diffusive signal. The signal does not diffuse within the field occupied by the cells. Cells absorb the signal from adjacent regions and determine their polarity strength while consuming the signal. The color of the field represents the amount of signal, and the color of the arrows represents the strength of each cell’s polarity. For details of the model, refer to Section 4 in S1 Appendix.

The early embryos of mammals initially form a solid mass, which then differentiates into an outer monolayer sphere and an inner cell mass. This behavior can be reproduced by allowing *p* to change over time in response to an external signal. As shown in Fig 11, the outer cells are exposed to the signal and acquire polarity, exhibiting monolayer inflation behavior. Whereas the outer cells acquire polarity, the inner cells cannot receive the signal because the outer cells act as a barrier, so they keep low polarity and stay in the random mass phase. This corresponds to the characteristic morphogenetic event in early mammalian development, where outer cells organize into a single-layered epithelium surrounding an inner cell mass that remains inside.

## 4. Discussion

In this study, we used a mathematical model to investigate the role of polarity in the formation of cellular layers, masses, and cavities. Despite its simplicity, our model incorporating only adhesion and polarity is able to replicate the basic types of morphogenetic patterns observed in early embryogenesis. A key feature of the model is that the observed patterns are governed by just two parameters. The first one is *p*, which represents the strength of polarity and thereby controls the extent to which polarity drives structural organization. The second one is $\tau_{B}$, which specifies the time scale of polarity regulation, i.e., how rapidly polarity orientations change; in our analysis, $\tau_{V}$ was fixed and $\tau_{B}$ was varied to assess its effect. By focusing on these two parameters, the diversity of morphogenetic behaviors is generated by the model, as can be systematically understood. Our findings revealed that the number of cellular layers decreases with the polarity parameter *p*, transitioning from multilayer to monolayer configurations. Additionally, we identified two distinct mechanisms of cavity formation: wraparound and inflation. The mode of cavity formation is determined by the time scale of polarity regulation ($\tau_{B}$), highlighting the critical role of polarity regulation in cavity development within multicellular systems. Overall, our model provides a theoretical framework for understanding the morphogenesis of multicellular systems and offers insights into how polarity and its regulation contribute to the structural complexity observed in real organisms.

Our model highlights the importance of the time scale of polarity regulation in determining the mode of cavity formation. This effect described by the second term in the Equation (7) is based on the assumption that cell adhesion influences the orientation of polarity. In fact, examples of polarity formation observed in various developmental processes [34,35] and organoids [36] clearly indicate that adhesion exerts some control over polarity, typically by orienting the apical side distinct from adhesion sites. Given these biological observations, we consider our modeling assumption to be qualitatively natural. At the same time, we emphasize that Eq. (7) is not intended to represent a molecularly explicit polarity-establishment pathway; rather, it is a minimal phenomenological rule for coupling adhesive or mechanical context to polarity orientation. Fundamental morphogenetic patterns can be understood as generic physical processes [9], in the spirit of universal biology [37].

The parameter *p* should be interpreted as a coarse-grained measure of how strongly polarity generates an adhesive differential between more-adhesive and less-adhesive regions of the cell surface. Likewise, $\tau_{B}$, although introduced as a timescale parameter, can be interpreted as an effective measure of the degree to which neighboring cells reorient their apico-basal polarities away from each other and from adhesive interfaces in response to adhesive or mechanical cues. In this sense, larger $\tau_{B}$ corresponds to a stronger tendency for adjacent cells to bias their polarities outward relative to the local adhesive surface. In experimental systems, these effective parameters could in principle be modulated by perturbing cadherin-mediated adhesion, cortical tension, or mechanosensitive pathways. Such perturbations may alter the adhesive differential across the cell surface or the extent to which cell-cell contact and mechanical stress bias apico-basal polarity orientation, thereby shifting the morphology across phase boundaries and potentially changing layer number and cavity-formation mode.

In real developmental systems, microscopic parameters such as polarity strength and the timescale of polarity regulation are expected to vary from cell to cell, rather than being exactly identical. Although the main phase diagram in this study was obtained for the homogeneous model, additional simulations with small cell-to-cell heterogeneity in *p* or $\tau_{B}$ showed that the representative morphologies are qualitatively preserved. This suggests that the phase diagram reported here is not only for the case with uniform cells but also remains robust under modest biological variability.

In this model, qualitative differences in macroscopic morphogenesis can be explained by the combination of only two microscopic parameters: the polarity strength *p* and the time scale of angle change $\tau_{B}$. As shown in Fig 6, distinct morphogenetic modes, such as multilayer inflation and monolayer wraparound, emerge in adjacent regions of the $\left(p,\tau_{B}\right)$ parameter space. This indicates that just a change in these parameters can trigger a drastic transition in the global morphogenetic behavior. Consequently, when interpreting evolutionary changes in developmental processes through this model, similarity in macroscopic morphology may not necessarily correspond to phylogenetic proximity. This result from the model is consistent with observations that amphibians and mammals generally exhibit inflation-like morphogenesis in the early embryogenesis, whereas many fish (including zebrafish) and birds typically show wraparound-like patterns.

Our results provide a new perspective on the role of polarity in morphogenesis, which can be validated and observed through experiments. By effectively altering the parameters *p* and $\tau_{B}$, it may be possible to change the mode of morphogenesis, according to the phase diagram in Fig 6. For example, cadherins are known to mediate cell-cell adhesion and play a critical role in maintaining tissue integrity and structure [12]. By manipulating the expression levels of cadherins, researchers have observed changes in tissue morphology, supporting the idea that cell adhesion strength can influence macroscopic morphology [13,38]. Furthermore, when E-cadherin is knocked out, mouse ES cells form a monolayer sphere by wrapping around, corresponding to the monolayer wraparound phase in our study; otherwise, they typically form a monolayer sphere by inflating from within during proliferation, which corresponds to the monolayer inflation phase [39,40]. This can be interpreted as a transition from the monolayer inflation phase to the monolayer wraparound phase in our study. By manipulating the expression levels or activity of such adhesion molecules, one could potentially influence the polarity dynamics and the resulting morphological outcomes. This suggests that the regulation of *p* and $\tau_{B}$ could serve as a mechanism to control the formation of different tissue structures or the number of layers, providing a valuable tool for both developmental biology and tissue engineering. In future studies, a more extensive exploration of the parameters will be necessary.

A limitation of the present model is that all interactions are pairwise local cell–cell interactions governed by the same polarity-dependent potential. In particular, the model does not include a separate sheet–sheet adhesion term. Therefore, the multilayer states reported here should not be interpreted as a detailed one-to-one model of stratified epithelia or multilaminar tissues with explicit inter-sheet mechanics. Rather, they are minimal outcomes of the same local rules in a regime where cells stack into more than one layer.

Here, we considered a model where the total number of cells increases linearly, but of course this is not always the case in reality. For example, if each cell divides with a constant cell cycle, the number of cells will increase exponentially. In this scenario, the number of new cells added to the system also increases exponentially, meaning that after a certain period, new cells are added before the adhesion dynamics have converged to a stable state. This can lead to phenomena such as buckling later, which differ from the results observed in this study. This type of dynamics that occur when cell proliferation is rapid are beyond the scope of this research. This limitation is related to previous studies of nonequilibrium epithelial growth. Cerruti et al. reported that when cell division is much faster than mechanical rearrangement and lumen dynamics, epithelial growth can deviate from equilibrium and generate aberrant multilumen phenotypes [30]. Our study addresses a complementary regime, slower growth conditions under which polarity-dependent adhesion and polarity regulation can be examined as the primary determinants of morphology. Extending the present model to faster division cases, and to incorporate lumen-specific mechanisms, will be an important direction for future work. The reason why the number of layers changes in this model is not yet explained analytically. It seems that, in principle, any number of layers could be stacked if the three-cell system possesses a stable fixed point that allows a third cell to pile on top (Fig 8c). However, our simulations show that the actual number of layers is limited and is determined at the macroscopic level rather than solely by local three-cell interactions. As a hypothesis, the number of layers may depend on the relative sizes of the basins of attraction corresponding to different stable configurations in the three-cell system. In other words, the probability that a third cell stabilizes on top of or within the existing layer may determine the macroscopic number of layers. Clarifying this relationship remains a topic for future research. Additionally, we were unable to analytically understand the boundary between wraparound and inflation in the multilayer case, which also depends on the value of *p*. This result remains a subject for future theoretical research as well.

An important corollary of the phase diagram approach is that it identifies possible constraints or prohibitions on morphological transitions. While the model predicts a possible repertoire of morphological processes, the topological arrangement of these phases suggests pathways that are not allowed under continuous variations in the morphogenetic parameters, for example through gradual genetic mutations or continuous experimental perturbations of *p* and $\tau_{B}$. A direct transition between non-adjacent phases in the diagram is not possible without passing through intermediate morphologies. For instance, transitioning from monolayer inflation to multilayer inflation requires passing through the intermediate monolayer wraparound. Bypassing the intermediate states would require large discontinuous changes in morphogenetic mechanisms beyond the minimal local rules described here.

The potential applications of our model are not limited to the morphogenesis of current organisms. The insights gained from our study can be useful for designing artificial multicellular systems, such as organoids, artificial meat, and regenerative medicine. Organoids, which are three-dimensional structures derived from stem cells that mimic the architecture and function of real organs, have become valuable tools for studying development and disease [41–43]. By applying our model to organoid systems, one can identify optimal culture conditions and strategies for manipulating cell polarity to achieve desired tissue structures. The possible and prohibited transitions discussed above may also be relevant to the design of organoid morphogenesis.

## Supporting information

S1 Appendix Supporting information. This file contains supplementary analyses and model details, including robustness tests against cell-to-cell heterogeneity, definitions of the quantitative measures used for morphology classification, three-dimensional examples of the model, and the detailed information of the model used for Fig 11.
(PDF)

S1 File References. (DOCX)
