# Supplementary material for: Adhesion and polarity-driven morphogenesis: Mechanisms and constraints in tissue formation
Source: PLoS Comput Biol. 2026 Jun 22;22(6):e1013939. doi: 10.1371/journal.pcbi.1013939 (PMC13286278; doi:10.1371/journal.pcbi.1013939)
Supplement: S1 File — (DOCX) [file pcbi.1013939.s002.docx]

31. Similar analysis would be possible for the multilayer case, but it is difficult to get analytical results.

32. If *ψ*_1_ that satisfies the second equation for cos *ψ*_1_ in Eq. (15) exists, that solution is stable.

33. In this model, boundary conditions are set to be circle (gray area in Fig 10). This is because, in two dimensions, the shape of the multilayer wrap­around does not robustly form a perfect circle. If the shape does not form a perfect circle, the effect of the gradient in τ_B_ can differ at both ends, causing inconsistencies. However, in the three-dimensional model, the multilayer wraparound often forms a more perfect sphere. Therefore, we consider this change in boundary conditions to be addressing a two-dimensional specific issue and not problematic.
