# Supplementary material for: Adhesion and polarity-driven morphogenesis: Mechanisms and constraints in tissue formation
Source: PLoS Comput Biol. 2026 Jun 22;22(6):e1013939. doi: 10.1371/journal.pcbi.1013939 (PMC13286278; doi:10.1371/journal.pcbi.1013939)
Supplement: Attachment — Submitted filename: Response_to_reviewers.pdf [file pcbi.1013939.s003.pdf]

Response to Reviewers

Manuscript ID: PCOMPBIOL-D-26-00154

Title: Adhesion and polarity-driven morphogenesis: Mechanisms and constraints in tissue formation

Reply to Reviewer #1

We would like to thank the reviewer for the positive evaluation, constructive criticism, and helpful comments to improve our manuscript. We have revised the manuscript following these suggestions. Our responses to the reviewer's comments are shown below.

Comment 1: What is T in equation 1?

Response 1: We agree that the notation in Eq. (1) requires clarification. The superscript "T" denotes vector transpose, so that $\boldsymbol{p}_i = (p\cos\theta_i,\, p\sin\theta_i)^{\mathrm{T}}$ is written as a column vector. We have stated this explicitly when Eq. (1) is introduced.

Revision 1: Model (line 109): "Here, the superscript T denotes transpose."

Comment 2: Why does equation 3 have a +1 term?

Response 2: The +1 term in Eq. (3) ensures that the polarity-dependent factor S_{ij} remains non-negative (given that we investigate the parameter range $p \leq 1$ in this study) and modulates the magnitude of adhesion without changing its sign. Without this offset, the polarity term could become negative for some relative orientations, which would qualitatively alter the interaction by effectively turning the adhesive contribution into a repulsive one. Our intention is that polarity changes the relative adhesive strength between different cell–cell interfaces, from weakly adhesive to strongly adhesive, while the baseline adhesive interaction remains attractive.

Revision 2: Model (line 131): "The offset term +1 in Eq. (3) provides a baseline contribution to adhesion and, since we restrict the polarity strength to $p \leq 1$ in this study, keeps the polarity-dependent factor S_{ij} non-negative. As a result, polarity modulates the strength of adhesion between cells, rather than changing the interaction from attractive to repulsive. In this sense, S_{ij} describes a difference in adhesive strength between more and less adhesive regions of the cell surface."

Comment 3: "On the other hand, the polarity θ_i is controlled not only by the potential V_i but also by adhesion." Can the authors give a rationale for this?

Response 3: We agree that the rationale should be stated more clearly. The second term in Eq. (7) is a coarse-grained representation of the tendency for apico-basal polarity to orient with respect to adhesive or mechanically stressed interfaces. In the model, this term biases the polarity to orient away from adhesive surfaces, thereby producing an apical/non-adhesive side and a basal/adhesive side at the coarse-grained level. We emphasize that this is not meant as a detailed molecular model of polarity establishment, but as an effective phenomenological coupling between adhesion and polarity orientation. We have revised the corresponding parts of the Model and Discussion sections to clarify this point, as shown below.

Revision 3: Model (line 158): "This term should be understood as a phenomenological polarity-reorientation rule. It captures the tendency for the apico-basal polarity to become

oriented away from adhesive interfaces.”

Revision 3: Discussion (line 430): “At the same time, we emphasize that Eq. (7) is not intended to represent a molecularly explicit polarity-establishment pathway; rather, it is a minimal phenomenological rule for coupling adhesive or mechanical context to polarity orientation.”

Comment 4: “Several experimental results support the fact that apico-basal polarity is controlled by mechanical stress and is often oriented perpendicular to the adhesion surface [18, 19]." This is true of simple epithelia but not of stratified or multilaminar epithelia: do the authors implement any adhesion between cell sheets in multilaminar structures.

Response 4: This is an important point. In the present model, there is no separate sheet-level adhesion term between already formed epithelial sheets. All interactions are pairwise cell–cell interactions governed by the same polarity-dependent adhesion potential. Therefore, the multilayer structures in our simulations should be interpreted as outcomes of the same local rules applied in a regime where cells stack into multiple layers, rather than as a dedicated model of stratified epithelia with distinct inter-sheet adhesion mechanisms. We have clarified this limitation and avoided overgeneralizing the biological interpretation. We have revised the Discussion section to clarify this limitation and avoid overgeneralizing the biological interpretation, as follows.

Revision 4: Discussion (line 485): “A limitation of the present model is that all interactions are pairwise local cell–cell interactions governed by the same polarity-dependent potential. In particular, the model does not include a separate sheet–sheet adhesion term. Therefore, the multilayer states reported here should not be interpreted as a detailed one-to-one model of stratified epithelia or multilaminar tissues with explicit inter-sheet mechanics. Rather, they are minimal outcomes of the same local rules in a regime where cells stack into more than one layer.”

Comment 5: In the manuscript the authors attribute the ability to give rise to morphogenetic diversity through: two microscopic parameters: the magnitude of polarity p and the time scale of angle change τ_B. Do these two terms, especially the second one have biological equivalents? How would it be possible to test the predictions of the model empirically. This should be alluded to in the discussion section.

Response 5: We agree that the biological interpretation of these parameters and the empirical testability of the model are crucial points. As detailed in the revised Discussion, we have clarified that p represents the magnitude of polarity-driven adhesion anisotropy—effectively the adhesive differential or ratio between more-adhesive and less-adhesive regions of the cell surface. Similarly, τ_B is interpreted as an effective measure of the degree to which neighboring cells reorient their apico-basal polarities away from each other and from adhesive interfaces in response to adhesive or mechanical cues.

Regarding empirical testing, we suggest that these parameters could, in principle, be modulated by perturbing molecules such as E-cadherin or polarity complexes. While the current model is a minimal one, it may provide a framework to explore how such perturbations could lead to shifts between different morphogenetic modes, such as the inflation- and wraparound-type cavity formation observed in our simulations. We have updated the Discussion to include these biological interpretations and to mention these potential avenues for experimental comparison.

Revision 5: Discussion (line 435): "The parameter p should be interpreted as a coarse-grained measure of how strongly polarity generates an adhesive differential between more-adhesive and less-adhesive regions of the cell surface. Likewise, although introduced phenomenologically, τ_B can be interpreted as an effective measure of the degree to which neighboring cells reorient their apico-basal polarities away from each other and from adhesive interfaces in response to adhesive or mechanical cues. In this sense, larger τ_B corresponds to a stronger tendency for adjacent cells to bias their polarities outward relative to the local adhesive surface. In experimental systems, these effective parameters could in principle be modulated by perturbing cadherin-mediated adhesion, cortical tension, or mechanosensitive pathways. Such perturbations may alter the adhesive differential across the cell surface or the extent to which cell-cell contact and mechanical stress bias apico-basal polarity orientation, thereby shifting the morphology across phase boundaries and potentially changing layer number and cavity-formation mode."

Comment 6: I may have missed it but the coauthors may not have worked with the possibility that tiny variations (or errors) in adhesion strength or polarity strength or its regulation timing. It is unlikely each cell should have equal magnitudes of these metrics. Would the morphogenesis be robust to small sets of values of such metrics rather than single values per simulation? If not addressed, this could be discussed by the authors.

Response 6: We thank the reviewer for raising this important point. We agree that individual cells in biological systems are unlikely to have exactly identical values of parameters such as the magnitude of polarity-dependent adhesion anisotropy or the timescale of polarity regulation.

To address this issue, we performed additional simulations with small cell-to-cell heterogeneity. Specifically, we assigned the parameter p or τ_B to each cell following a log-normal distribution (so that the logarithm of the parameter is normally distributed in the log-scaled parameter space). To demonstrate that the observed morphologies are robust even against variations of a meaningful magnitude, we set the standard deviation of this distribution to 10% of the grid spacing used in the phase diagram.

The representative time-series behaviors were qualitatively unchanged from the homogeneous simulations shown in Fig. 4. We therefore added these results as supplementary figures, using the same four-panel time-series format as Fig. 4. These results suggest that the morphogenetic behaviors reported in the main text are robust to modest cell-to-cell variability in either p or τ_B.

We examined two cases separately: one in which p varied among cells while τ_B was fixed, and another in which τ_B varied among cells while p was fixed. In both cases, the representative time-series behaviors were qualitatively unchanged from the homogeneous simulations shown in Fig. 4. We added these results as supplementary figures, using the same four-panel time-series format as Fig. 4. These results suggest that the morphogenetic behaviors reported in the main text are robust to such biologically plausible, modest cell-to-cell variability.

Revision 6: Results (line 233) / Supporting Information (Section 1 in S1 Appendix): "In the analyses above, we mainly considered the homogeneous case, in which all cells share the same parameter values. To examine whether the observed morphologies are robust to cell-to-cell variations, we also simulated a heterogeneous version of the model where the parameter p or τ_B varied across cells. Specifically, the parameter value for each cell was drawn from a log-normal distribution, where the center was set to the baseline value of the homogeneous simulation,

and the standard deviation was set to 10% of the grid size of the phase diagram. Under this heterogeneity, the representative behaviors corresponding to those in Fig. 4 were qualitatively unchanged (see details in Section 1 in S1 Appendix). These results indicate that the basic morphogenetic types described here are robust to modest cell-to-cell variability."

Revision 6: Discussion (line 448): "In real developmental systems, microscopic parameters such as polarity strength and the timescale of polarity regulation are expected to vary from cell to cell, rather than being exactly identical. Although the main phase diagram in this study was obtained for the homogeneous model, additional simulations with small cell-to-cell heterogeneity in p or τ_B showed that the representative morphologies are qualitatively preserved. This suggests that the phase structure reported here is not a fine-tuned artifact of perfectly uniform cells, but remains relevant under modest biological variability."

Reply to Reviewer #2

We would like to thank the reviewer for the thoughtful and constructive comments, especially the suggestions about historical context, terminology, and nonequilibrium aspects. We have revised the manuscript following these suggestions. Our responses are shown below.

Comment 1: The idea that cell adhesion and cell polarity, acting together, can give rise to cavities within tissues – a feature of animal embryogenesis, and in vitro models – is not a new one. If cells are sticky over their surfaces except for one region (by apicobasal polarization) and the cells readjust to minimize free energy, the equilibrium configuration would cause the non-adhesive domains to surround an interior space. This idea, perhaps first stated in (1), does not require a detailed model to be persuasive in its simplest form, and has been part of discussions about the evolution of animal body plans for several decades. (This could be made clearer in the introduction.)

Response 1: We agree with the reviewer. The basic idea of polarity-plus-adhesion-driven cavity formation is not new, but rather we intend to demonstrate how a minimal model gives rise to multiple morphogenetic outcomes that can be organized into a phase diagram. We have revised the Introduction to acknowledge the earlier conceptual literature more clearly.

Revision 1: Introduction (line 52): "Conceptually, the premise that cell adhesion and apico-basal polarity act together to give rise to cavities within tissues has been recognized; for instance, earlier physico-evolutionary arguments demonstrated that a hollow configuration naturally emerges from adhesive packing if cells are less adhesive on one polarized side [11]. Building on this foundation, several ..."

Comment 2: Several places in the text the authors refer to both adhesive strength and "polarity strength," as well as temporal regulation of polarity. They characterize their results, however, as being controlled by just the two polarity parameters. From the context, polarity strength is polarity-dependent adhesion, but this usage is not typical for biologists, who would find both this and "polarity strength" confusing. A key result of this paper is the effect of the ratio between the adhesive and less adhesive portions of the individual cells (i.e., in contrast to the all-or-none model). The idea of adhesive differential or ratio should be included to help visualize why or why not an interior space will form.

Response 2: We thank the reviewer for this helpful comment. We agree that the meaning of "polarity strength" in our model may not have been sufficiently clear to readers in biology. We have retained the term "polarity strength" as a shorthand, but we have clarified this point in the Model section. Regarding the terminology, we have also unified the various expressions (e.g., 'magnitude of polarity', 'strength of polarity') into 'polarity strength' to improve readability.

Specifically, we now state explicitly that, in our formulation, polarity acts through adhesion: the parameter p controls how strongly adhesion depends on the orientation of cell-cell contact relative to polarity. In other words, p can be interpreted as setting the contrast between more adhesive and less adhesive portions of the cell boundary. We also note that, because the polarity-dependent factor S_{ij} ranges from $1-p^2$ to $1+p^2$, this can be viewed as an adhesive differential (or ratio).

To make this interpretation easier to visualize, we have added a new Fig. 3b to indicate the more adhesive and less adhesive portions of the cell boundary using a color gradient and have added double-headed arrows to represent the strength of S_{ij}. We agree that this point is important for understanding why the formation of an interior space depends on the degree of adhesion contrast.

Revision 2: Model (line 120): "In this model, p sets the adhesive differential between the most and least adhesive parts of the cell surface, as illustrated in Fig. 3b. For simplicity, we refer to this parameter p as "polarity strength" throughout this manuscript. The factor S_{ij} ranges from $1 - p^2$ to $1 + p^2$, depending on the relative orientation between the polarity axis and the cell-cell contact. This interpretation is useful for understanding how changes in p affect the formation of cavities, which can be understood as a competition between the gain in adhesive energy and the cost of maintaining non-adhesive surfaces. When p is large, the adhesive differential is high enough that the cells prefer to maximize contacts at their highly adhesive lateral sides. This radial alignment creates a "non-adhesive core" that prevents the collapse of the structure into a solid mass, thereby stabilizing a central cavity."

Revision 2: Modified Figure 3

Comment 3: It seems that the described model is a quasi-equilibrium one, in that energy-minimizing interactions among cells lead to alignment of polarities, and this produces different morphologies, depending on values of the two control parameters. The addition of new cell by growth does not affect these assumptions. However, a 2013 paper (2), not cited here, argues that depending or the ratio of cell division rate to the parameters of Potts-based model for lumen formation, effects of nonequilibrium dynamics such as multilumens, appear. The present model would benefit from engaging with the results of Cerruti et al., possibly by introducing variation in proliferation rate.

Response 3: We thank the reviewer for pointing out the important work by Cerruti et al. (2013). We have now added this reference and clarified the relation between their study and ours.

Cerruti et al. demonstrated that, in a 3D Potts-based model of epithelial cyst growth, the ratio between cell-division rate and mechanical/lumen-relaxation timescales can drive the system far from equilibrium and induce aberrant multilumen phenotypes. In contrast, the purpose of the present study is to characterize the repertoire of morphologies that arise from polarity-dependent adhesion in the regime where positional and polarity rearrangements are sufficiently fast relative to cell division. In this sense, our analysis is intentionally focused on the slow-

growth / quasi-steady regime.

We agree that proliferation-rate dependence is an important extension. However, in the current minimal model, very rapid division primarily leads to generic crowding-driven effects such as mass formation or buckling, which are out of the main scope of the present work. Because our model does not include explicit lumen-specific processes such as apical membrane initiation, lumen pressure, or spindle-orientation control, we believe that a detailed comparison to the multilumen mechanism studied by Cerruti et al. would require substantial extensions of the model and would go beyond the central aim of this paper.

To address the reviewer's concern, we have revised the manuscript to: (i) cite Cerruti et al. (2013), (ii) explicitly state that our phase diagram should be interpreted as applying to the slow-growth regime, and (iii) discuss nonequilibrium proliferation-induced multilumen formation as an important future direction.

Revision 3: Introduction (line 62): "Previous theoretical studies have also highlighted the importance of nonequilibrium growth in epithelial morphogenesis. In particular, Cerruti et al. used a three-dimensional Potts-based model and experiments to show that the ratio between cell division and mechanical/lumen relaxation timescales determines whether epithelial growth proceeds close to equilibrium or far from it, and that rapid nonequilibrium growth can induce aberrant multilumen phenotypes [30]. In the present study, by contrast, we focus on the complementary regime in which cell division is sufficiently slow compared with positional and polarity relaxation, in order to extract the morphology-selection mechanism attributable specifically to polarity-dependent adhesion."

Revision 3: Model (line 180): "Accordingly, the present analysis should be interpreted as applying to a slow-growth regime, in which local positional and polarity relaxation substantially proceeds before cell division. Our aim is to extract the morphology-selection mechanism due to polarity-dependent adhesion rather than nonequilibrium effects induced by excessively rapid proliferation. "

Revision 3: Discussion (line 500): "This limitation is related to previous studies of nonequilibrium epithelial growth. Cerruti et al. reported that when cell division is much faster than mechanical rearrangement and lumen dynamics, epithelial growth can deviate from equilibrium and generate aberrant multilumen phenotypes [30]. Our study addresses a complementary regime, slower growth conditions under which polarity-dependent adhesion and polarity regulation can be examined as the primary determinants of morphology. Extending the present model to faster-division cases and incorporating lumen-specific mechanisms, will be an important direction for future work."

Comment 4: Based on the model's assumptions there should be not only predictions of possible morphologies (as provided) but also prohibited states. If any of these can be specified, they would be of interest to experimental developmental biologists (particularly if the prohibitions could be relieved by genetic manipulations), and evolutionary biologists.

Response 4: We thank the reviewer for this insightful perspective. As the reviewer points out, identifying what our results precludes is important. While it is challenging to strictly prove the absolute impossibility of certain tissue shapes across all conceivable parameter regimes, our phase diagram clearly reveals "prohibited transitions" and constraints in developmental or evolutionary pathways.

Because the morphologies are organized into distinct, contiguous phases in the parameter space,

continuous changes in biological parameters (e.g., through gradual evolutionary mutations or dose-dependent experimental perturbations of adhesion/polarity) dictate specific transition routes. For instance, transitioning from "monolayer inflation" to "multilayer inflation" requires passing through the intermediate "monolayer wraparound". A direct transition between non-adjacent phases is thus "prohibited" under continuous parameter variations. We have added a new paragraph to the Discussion to highlight the relevance of these topological constraints to evolution and development.

Revision 4: Discussion (line 521): "An important corollary of the phase diagram approach is that it identifies possible constraints or prohibitions on morphological transitions. While the model predicts a possible repertoire of morphological processes, the topological arrangement of these phases suggests pathways that are not allowed under continuous variations in the morphogenetic parameters, for example through gradual genetic mutations or continuous experimental perturbations of p and τ_B. A direct transition between non-adjacent phases in the diagram is not possible without passing through intermediate morphologies. For instance, transitioning from monolayer inflation to multilayer inflation requires passing through the intermediate monolayer wraparound. Bypassing the intermediate states would require large discontinuous changes in morphogenetic mechanisms beyond the minimal local rules described here."
