# Supplementary material for: Adhesion and polarity-driven morphogenesis: Mechanisms and constraints in tissue formation
Source: PLoS Comput Biol. 2026 Jun 22;22(6):e1013939. doi: 10.1371/journal.pcbi.1013939 (PMC13286278; doi:10.1371/journal.pcbi.1013939)
Supplement: Peer review history

# Peer review: Decision Letter 0

**DOI:** 10.1371/journal.pcbi.1013939.r001

**Authors:** Attila Csikász-Nagy, Stacey Finley

25 Mar 2026

PCOMPBIOL-D-26-00154

Adhesion and polarity-driven morphogenesis: Mechanisms and constraints in tissue formation

PLOS Computational Biology

Dear Dr. Furusawa,

Thank you for submitting your manuscript to PLOS Computational Biology. After careful consideration, we feel that it has merit but does not fully meet PLOS Computational Biology's publication criteria as it currently stands. Therefore, we invite you to submit a revised version of the manuscript that addresses the points raised during the review process.

Please submit your revised manuscript by May 25 2026 11:59PM. If you will need more time than this to complete your revisions, please reply to this message or contact the journal office at ploscompbiol@plos.org. When you're ready to submit your revision, log on to https://www.editorialmanager.com/pcompbiol/ and select the 'Submissions Needing Revision' folder to locate your manuscript file.

Please include the following items when submitting your revised manuscript:

* A letter that responds to each point raised by the editor and reviewer(s). You should upload this letter as a separate file labeled 'Response to Reviewers'. This file does not need to include responses to formatting updates and technical items listed in the 'Journal Requirements' section below.

* A marked-up copy of your manuscript that highlights changes made to the original version. You should upload this as a separate file labeled 'Revised Manuscript with Track Changes'.

* An unmarked version of your revised paper without tracked changes. You should upload this as a separate file labeled 'Manuscript'.

If you would like to make changes to your financial disclosure, competing interests statement, or data availability statement, please make these updates within the submission form at the time of resubmission. Guidelines for resubmitting your figure files are available below the reviewer comments at the end of this letter

We look forward to receiving your revised manuscript.

Kind regards,

Attila Csikász-Nagy

Academic Editor

PLOS Computational Biology

Stacey Finley

Section Editor

PLOS Computational Biology

**Additional Editor Comments:**

The reviewers raise significant points that the authors should address, including details regarding the approach and placing this work in context and addressing relevant related work.

**Journal Requirements:**

1) Please ensure that the CRediT author contributions listed for every co-author are completed accurately and in full.

At this stage, the following Authors/Authors require contributions: Yoshiyuki T Nakamura, Chikara Furusawa, and Kunihiko Kaneko. Please ensure that the full contributions of each author are acknowledged in the "Add/Edit/Remove Authors" section of our submission form.

The list of CRediT author contributions may be found here: https://journals.plos.org/ploscompbiol/s/authorship#loc-author-contributions

2) We ask that a manuscript source file is provided at Revision. Please upload your manuscript file as a .doc, .docx, .rtf or .tex. If you are providing a .tex file, please upload it under the item type u2018LaTeX Source Fileu2019 and leave your .pdf version as the item type u2018Manuscriptu2019.

3) Please upload all main figures as separate Figure files in .tif or .eps format. For more information about how to convert and format your figure files please see our guidelines:

https://journals.plos.org/ploscompbiol/s/figures

4) We have noticed that you have uploaded Supporting Information files, but you have not included a list of legends. Please add a full list of legends for your Supporting Information files after the references list.

5) Please amend your detailed Financial Disclosure statement. This is published with the article. It must therefore be completed in full sentences and contain the exact wording you wish to be published.

State what role the funders took in the study. If the funders had no role in your study, please state: "The funders had no role in study design, data collection and analysis, decision to publish, or preparation of the manuscript.".

If you did not receive any funding for this study, please simply state: u201cThe authors received no specific funding for this work.u201d

**Reviewers' comments:**

Reviewer's Responses to Questions

**Comments to the Authors:**

**Please note here if the review is uploaded as an attachment.**

Reviewer #1: The manuscript titled "Adhesion and polarity-driven morphogenesis: Mechanisms and constraints in tissue formation" by Nakamura and coworkers is a timely and elegant manuscript exploring the morphogenesis of diverse forms through cellular properties: polarity and adhesion. The manuscript is very well written and lucid and I have a few minor comments:

1: what is T is equation 1?

2: why does equation 3 have a +1 term?

3: "On the other hand, the polarity θi is controlled not only by the potential Vi but also by adhesion." Can the authors give a rationale for this?

4: "Several experimental results support the fact that apico-basal polarity is controlled by mechanical stress and is often oriented perpendicular to the adhesion surface [18, 19]." This is true of simple epithelia but not of stratified or multilaminar epithelia: do the authors implement any adhesion between cell sheets in multilaminar structures.

5: In the manuscript the authors attribute the ability to give rise to morphogenetic diversity through: two microscopic parameters: the magnitude of polarity p and the time scale of angle change τB.

do these two terms, especially the second one have biological equivalents? How would it be possible to test the predictions of the model empirically. This should be alluded to in the discussion section.

6: I may have missed it but the coauthors may not have worked with the possibility that tiny variations (or errors) in adhesion strength or polarity strength or its regulation timing. It is unlikely each cell should have equal magnitudes of these metrics. Would the morphogenesis be robust to small sets of values of such metrics rather than single values per simulation? If not addressed, this could be discussed by the authors.

Reviewer #2: The idea that cell adhesion and cell polarity, acting together, can give rise to cavities within tissues – a feature of animal embryogenesis, and in vitro models – is not a new one. If cells are sticky over their surfaces except for one region (by apicobasal polarization) and the cells readjust to minimize free energy, the equilibrium configuration would cause the non-adhesive domains to surround an interior space. This idea, perhaps first stated in (1), does not require a detailed model to be persuasive in its simplest form, and has been part of discussions about the evolution of animal body plans for several decades. (This could be made clearer in the introduction.)

The problem with the simple, intuitive idea, however, is that it represents an all-or-none model (regarding both adhesion and polarity) and is based on an equilibrium argument. Biological phenomena are rarely so binary, and these assumptions may break down. This is why mathematical and computational models like that presented here are essential to a comprehensive understanding of epithelial morphogenesis in the development of natural embryos, in comparative evolutionary biology, and in in vitro organoids. The phase diagrams provided are particularly helpful.

The following are considerations that I believe need to be addressed or better clarified to make this paper fully suitable for publication in PLOS Computational Biology:

1) Several places in the text the authors refer to both adhesive strength and “polarity strength,” as well as temporal regulation of polarity. They characterize their results, however, as being controlled by just the two polarity parameters. From the context, polarity strength is polarity-dependent adhesion, but this usage is not typical for biologists, who would find both this and “polarity strength” confusing. A key result of this paper is the effect of the ratio between the adhesive and less adhesive portions of the individual cells (i.e., in contrast to the all-or-none model). The idea of adhesive differential or ratio should be included to help visualize why or why not an interior space will form.

2) It seems that the described model is a quasi-equilibrium one, in that energy-minimizing interactions among cells lead to alignment of polarities, and this produces different morphologies, depending on values of the two control parameters. The addition of new cell by growth does not affect these assumptions. However, a 2013 paper (2), not cited here, argues that depending or the ratio of cell division rate to the parameters of Potts-based model for lumen formation, effects of nonequilibrium dynamics such as multilumens, appear. The present model would benefit from engaging with the results of Cerruti et al., possibly by introducing variation in proliferation rate.

3) Based on the model’s assumptions there should be not only predictions of possible morphologies (as provided) but also prohibited states. If any of these can be specified, they would be of interest to experimental developmental biologists (particularly if the prohibitions could be relieved by genetic manipulations), and evolutionary biologists.

(1) Newman, S.A., 1998. Epithelial morphogenesis: a physico-evolutionary interpretation, in: Chuong, C.-M. (Ed.), Molecular Basis of Epithelial Appendage Morphogenesis. R. G. Landes, Austin, TX, pp. 341-358.

(2) Cerruti, B., Puliafito, A., Shewan, A.M., Yu, W., Combes, A.N., Little, M.H., Chianale, F., Primo, L., Serini, G., Mostov, K.E., Celani, A., Gamba, A., 2013. Polarity, cell division, and out-of-equilibrium dynamics control the growth of epithelial structures. J Cell Biol 203, 359-372.

**********

**Have the authors made all data and (if applicable) computational code underlying the findings in their manuscript fully available?**

The PLOS Data policy requires authors to make all data and code underlying the findings described in their manuscript fully available without restriction, with rare exception (please refer to the Data Availability Statement in the manuscript PDF file). The data and code should be provided as part of the manuscript or its supporting information, or deposited to a public repository. For example, in addition to summary statistics, the data points behind means, medians and variance measures should be available. If there are restrictions on publicly sharing data or code —e.g. participant privacy or use of data from a third party—those must be specified.

Reviewer #1: Yes

Reviewer #2: Yes

**********

PLOS authors have the option to publish the peer review history of their article (what does this mean?). If published, this will include your full peer review and any attached files.

If you choose “no”, your identity will remain anonymous but your review may still be made public.

**Do you want your identity to be public for this peer review?**           For information about this choice, including consent withdrawal, please see our Privacy Policy.

Reviewer #1: No

Reviewer #2: No

[NOTE: If reviewer comments were submitted as an attachment file, they will be attached to this email and accessible via the submission site. Please log into your account, locate the manuscript record, and check for the action link "View Attachments". If this link does not appear, there are no attachment files.]

**Figure resubmission:**

While revising your submission, we strongly recommend that you use PLOS’s NAAS tool (https://ngplosjournals.pagemajik.ai/artanalysis) to test your figure files. NAAS can convert your figure files to the TIFF file type and meet basic requirements (such as print size, resolution), or provide you with a report on issues that do not meet our requirements and that NAAS cannot fix.

After uploading your figures to PLOS’s NAAS tool - https://ngplosjournals.pagemajik.ai/artanalysis, NAAS will process the files provided and display the results in the "Uploaded Files" section of the page as the processing is complete. If the uploaded figures meet our requirements (or NAAS is able to fix the files to meet our requirements), the figure will be marked as "fixed" above. If NAAS is unable to fix the files, a red "failed" label will appear above. When NAAS has confirmed that the figure files meet our requirements, please download the file via the download option, and include these NAAS processed figure files when submitting your revised manuscript.

**Reproducibility:**

To enhance the reproducibility of your results, we recommend that authors of applicable studies deposit laboratory protocols in protocols.io, where a protocol can be assigned its own identifier (DOI) such that it can be cited independently in the future. Additionally, PLOS ONE offers an option to publish peer-reviewed clinical study protocols. Read more information on sharing protocols at https://plos.org/protocols?utm_medium=editorial-email&utm_source=authorletters&utm_campaign=protocols

# Author comment: Author response to Decision Letter 1

**DOI:** 10.1371/journal.pcbi.1013939.r002

27 May 2026

Attachment Submitted filename: Response_to_reviewers.pdf

# Peer review: Decision Letter 1

**DOI:** 10.1371/journal.pcbi.1013939.r003

**Authors:** Attila Csikász-Nagy, Stacey Finley, Attila Csikász-Nagy, Stacey Finley

1 Jun 2026

Dear Dr. Furusawa,

We are pleased to inform you that your manuscript 'Adhesion and polarity-driven morphogenesis: Mechanisms and constraints in tissue formation' has been provisionally accepted for publication in PLOS Computational Biology.

Before your manuscript can be formally accepted you will need to complete some formatting changes, which you will receive in a follow up email. A member of our team will be in touch with a set of requests.

Please note that your manuscript will not be scheduled for publication until you have made the required changes, so a swift response is appreciated.

IMPORTANT: The editorial review process is now complete. PLOS will only permit corrections to spelling, formatting or significant scientific errors from this point onwards. Requests for major changes, or any which affect the scientific understanding of your work, will cause delays to the publication date of your manuscript.

Should you, your institution's press office or the journal office choose to press release your paper, you will automatically be opted out of early publication. We ask that you notify us now if you or your institution is planning to press release the article. All press must be co-ordinated with PLOS.

Thank you again for supporting Open Access publishing; we are looking forward to publishing your work in PLOS Computational Biology.

Best regards,

Attila Csikász-Nagy

Academic Editor

PLOS Computational Biology

Stacey Finley

Section Editor

PLOS Computational Biology

***********************************************************

Reviewer's Responses to Questions

**Comments to the Authors:**

**Please note here if the review is uploaded as an attachment.**

Reviewer #2: The authors have addressed all the concerns raised in my review.

**********

**Have the authors made all data and (if applicable) computational code underlying the findings in their manuscript fully available?**

The PLOS Data policy requires authors to make all data and code underlying the findings described in their manuscript fully available without restriction, with rare exception (please refer to the Data Availability Statement in the manuscript PDF file). The data and code should be provided as part of the manuscript or its supporting information, or deposited to a public repository. For example, in addition to summary statistics, the data points behind means, medians and variance measures should be available. If there are restrictions on publicly sharing data or code —e.g. participant privacy or use of data from a third party—those must be specified.

Reviewer #2: Yes

**********

PLOS authors have the option to publish the peer review history of their article (what does this mean?). If published, this will include your full peer review and any attached files.

If you choose “no”, your identity will remain anonymous but your review may still be made public.

**Do you want your identity to be public for this peer review?**           For information about this choice, including consent withdrawal, please see our Privacy Policy.

Reviewer #2: No

# Editor report: Acceptance letter

**DOI:** 10.1371/journal.pcbi.1013939.r004

**Authors:** Attila Csikász-Nagy, Stacey Finley, Attila Csikász-Nagy, Stacey Finley

PCOMPBIOL-D-26-00154R1

Adhesion and polarity-driven morphogenesis: Mechanisms and constraints in tissue formation

Dear Dr Furusawa,

I am pleased to inform you that your manuscript has been formally accepted for publication in PLOS Computational Biology. Your manuscript is now with our production department and you will be notified of the publication date in due course.

The corresponding author will soon be receiving a typeset proof for review, to ensure errors have not been introduced during production. Please review the PDF proof of your manuscript carefully, as this is the last chance to correct any errors. Please note that major changes, or those which affect the scientific understanding of the work, will likely cause delays to the publication date of your manuscript.

Soon after your final files are uploaded, unless you have opted out, the early version of your manuscript will be published online. The date of the early version will be your article's publication date. The final article will be published to the same URL, and all versions of the paper will be accessible to readers.

For Research, Software, and Methods articles, you will receive an invoice from PLOS for your publication fee after your manuscript has reached the completed accept phase. If you receive an email requesting payment before acceptance or for any other service, this may be a phishing scheme. Learn how to identify phishing emails and protect your accounts at https://explore.plos.org/phishing.

Thank you again for supporting PLOS Computational Biology and open-access publishing. We are looking forward to publishing your work!

With kind regards,

Anita Estes

PLOS Computational Biology | Carlyle House, Carlyle Road, Cambridge CB4 3DN | United Kingdom ploscompbiol@plos.org | Phone +44 (0) 1223-442824 | ploscompbiol.org | @PLOSCompBiol
